# Mosaics of climatic stress across species' ranges: tradeoffs cause adaptive evolution to limits of climatic tolerance

**DOI:** 10.1098/rstb.2021.0003

**Published:** 2022-04-11

**Authors:** Camille Parmesan, Michael C. Singer

**Affiliations:** ^1^ Station d’Écologie Théorique et Expérimentale, CNRS, 2 route du CNRS, 09200 Moulis, France; ^2^ Biological and Marine Sciences, University of Plymouth, Plymouth PL4 8AA, UK; ^3^ Department of Geological Sciences, University of Texas at Austin, Austin, Texas 78712, USA

**Keywords:** range boundary, climate change, adaptation, geographic mosaic, thermal tolerance, phenological asynchrony

## Abstract

Studies in birds and trees show climatic stresses distributed across species' ranges, not only at range limits. Here, new analyses from the butterfly *Euphydryas editha* reveal mechanisms generating these stresses: geographic mosaics of natural selection, acting on tradeoffs between climate adaptation and fitness traits, cause some range-central populations to evolve to limits of climatic tolerance, while others remain resilient. In one ecotype, selection for predator avoidance drives evolution to limits of thermal tolerance. In a second ecotype, the endangered Bay Checkerspot, selection on fecundity drives evolution to the climate-sensitive limit of ability to complete development within the lifespans of ephemeral hosts, causing routinely high mortality from insect–host phenological asynchrony. The tradeoff between maternal fecundity and offspring mortality generated similar values of fitness on different dates, partly explaining why fecundity varied by more than an order of magnitude. Evolutionary response to the tradeoff rendered climatic variability the main driver of Bay Checkerspot dynamics, and increases in this variability, associated with climate change, were a key factor behind permanent extinction of a protected metapopulation. Finally, we discuss implications for conservation planning of our finding that adaptive evolution can reduce population-level resilience to climate change and generate geographic mosaics of climatic stress.

This article is part of the theme issue ‘Species’ ranges in the face of changing environments (Part II)’.

## 1. Introduction

Palaeontological records clearly document species responding to past climate changes with latitudinal range shifts [1]. More recently, in the first part of the twentieth century, prior to the current bout of anthropogenic warming, climate-caused range shifts occurred but were regional and temporary, in response to decadal temperature trends driven by known cyclical phenomona such as ENSO and NAO cycles, or by variation in solar activity [2,3]. Now that anthropogenic warming is well underway, increasing climatic stress at warm limits and decreasing stress at cool ones should engender range shifts that mitigate each species' experience of changing climate. This expectation is robustly and globally fulfilled: range shifts are occurring, and 80–92% of them have been in the directions expected from regional warming [4–13]. Within this generality, species' experiences of changing climate and their responses to that experience differ among biomes, habitats and organisms. Some systems align well with simple theories of how climatic stress should change across a species' range, while others respond in more complex ways, suggesting that climatic stress is not operating along predictable gradients.

Expanding range margins manifest complex and often unexpected eco-evolutionary phenomena [14–20], but in range centres we might expect simpler dynamics, with phenotypes of central populations changing gradually to resemble those of warmer regions under some combination of plasticity [21], selection and gene flow [22–26]. Such localized responses can take the form of changes in dispersal behaviour, voltinism, dietary specialization, camouflage, phenology or microhabitat choice [6,10,27].

In order to understand these responses, it is helpful to begin by considering communities that come closest to the straightforward theory of climate and species' range in which thermal stress is maximized at range extremes: stress associated with effects of hot climate increases monotonically towards range limits at low latitude/low altitude, while stress associated with cold climates increases towards limits at high latitude/high altitude. Individuals at cool range limits should be the most vulnerable to stress from prolonged cold temperatures or brief cold extremes, while the opposite should hold for warm limits. Under these assumptions, current range shifts should mostly involve local extinctions and range contractions at warm limits and expansions at cool margins.

Clearly, the communities that best match this theory are open-ocean. Oceanic species have tended to keep pace with warming temperatures, with 3/4 of species maintaining their species-specific climate envelopes [8,11,28–30]. These effects are sufficiently general that marine species richness is declining faster around the equator than at other latitudes [31]. Effects of light and oxygen are important in specific cases—for example, the need for high light intensity constrains poleward range shifts of tropical corals [32]—but these cases fail to mask the overarching influence of temperature on marine range dynamics.

By contrast, range dynamics in terrestrial and intertidal systems have been more diverse than in open-marine environments and less predictable from changes of regional temperature. There are several likely contributing factors to this difference:

(1) The velocity of climate change (e.g. geographic shifts in annual mean temperature isotherms over time) is greater in the ocean than on land and associated with greater observed range shifts in oceans than on land [28,29].
(2) Terrestrial ecotherms operate, on average, less close to their thermal limits than do their marine counterparts, so may be less immediately impacted by warming [30].
(3) Many terrestrial range limits are set by moisture rather than temperature [33]; precipitation can influence coastal marine species, but is typically more important to a tree than a shark.
(4) Cool thermal micro-refugia are more widespread and accessible to terrestrial, freshwater and intertidal organisms than to open-ocean species, although their availability decreases towards warm range limits [34].
(5) Human activities in terrestrial, intertidal and freshwater habitats create both thermal mosaics that diversify climatic stress and barriers to dispersal that physically impede range shifts [35–37].
(6) Changing elevation on land is not equivalent to changing depth in the ocean. Each system has its own specific non-temperature limitations stemming from oxygen, light and pressure gradients.

Presumably because of a combination of the marine/terrestrial differences that we list above, terrestrial species' ranges have been less immediately responsive to climate warming than their marine counterparts. Warm terrestrial range limits have been less mobile, tending to lag behind regional warming and apparently accumulating ‘climate debt’ [7,28,29,38]. In spite of these lags, hundreds of population extinctions along warm range boundaries have been related to recent climate change [39] and community-level effects of range shifts are becoming clear. For example, both richness and abundance of Californian butterflies have been decreasing at low and moderate elevations but increasing at the highest elevations [40].

Here, we begin with a brief review of intertidal, avian and botanical examples in which climatic stress has been demonstrated far from species' range limits, suggesting a generality of this phenomenon. We then use our study species, Edith's Checkerspot butterfly, to demonstrate underlying mechanisms driving this non-traditional pattern, showing how and why climatic stress is distributed patchily across the species' range, with local adaptation to hosts and habitats causing populations that are severely climate stressed to exist interdigitated with those that are not.

## 2. Geographic mosaics in climate-related stress—a non-exhaustive review

In the simple model with which we started, natural selection stemming from climatic stresses should increase monotonically towards range limits—especially warm limits under anthropogenic warming trends. We have already cited reasons why terrestrial and intertidal systems frequently violate that simple model. The fact that this violation results in geographic mosaics of climate stress through species' ranges, including range centres, was first recognized in a synthesis of intertidal studies [41]. With few exceptions [42–44], almost all subsequent references to climate stress mosaics concern intertidal systems in which stress mosaics are driven very directly by mosaics of temperature and moisture from which sessile organisms cannot escape (e.g. [45–47]).

**Figure 1.**
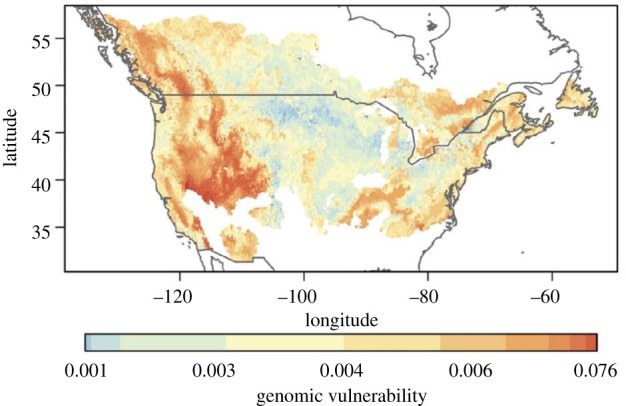
Map of climate stress in the yellow warbler. Expectations of local genotype were derived from local climatic data and overall association between genotype and climate across the range. ‘Genomic vulnerability’ at each site, used as an estimate of climatic stress, was derived as local deviation from climate-based expectation. The strongest outliers from the averaged statistical model prediction, shown by increasing rust colour, represent populations estimated from genomic data to be under most climatic stress. From Bay RA, Harrigan RJ, Le Underwood V *et al*. [44], Genomic signals of selection predict climate-driven population declines in a migratory bird. *Science* **359**, 83–86, reprinted with permission from AAAS.

Henceforth, we narrow our focus to the distribution of stress across ranges of terrestrial species. Our first two well-researched examples document climatic stresses in central sections of species' ranges. First, Bay *et al*. [44] showed that stresses were distributed in a mosaic across the geographic range of yellow warblers (*Setophaga petechia*) in North America. An overall association between genomic data and local climate was used to derive genomic signatures of climate adaptation. Climatic stresses, and the strengths of local natural selection on climate adaptations, were then deduced as population-level deviations from the genomic signatures expected from local climates. We show the geographic distribution of these stresses in figure 1: a complex picture, but clearly more of a mosaic than a cline, and with stress by no means confined to poleward and equatorial range margins. These estimates of local adaptation/maladaptation across the species' range were validated by using citizen science data to show that they were correlated with local population dynamic trends: there was a significant association between observed population declines and climatic stress estimated from genomic data [44].

Our second example is from Choat *et al*. [48, p. 752], who analysed measures of drought sensitivity in 226 tree species from 81 sites distributed globally. They found narrow ‘hydraulic safety margins’ in 70% of the study species, regardless of local climate. They expressed their findings thus:

‘Safety margins are largely independent of mean annual precipitation, showing that there is global convergence in the vulnerability of forests to drought, with all forest biomes equally vulnerable to hydraulic failure regardless of their current rainfall environment. These findings provide insight into why drought-induced forest decline is occurring not only in arid regions but also in wet forests not normally considered at drought risk’.

Drought stress clearly occurred in mesic range centres as well as at dry range limits. We perceive this finding as analogous with the results from Bay *et al*. [44] (figure 1), despite the two studies concerning very different organisms. Although Choat *et al*. [48] use modern data, these authors do not describe their results as effects of climate change and the patterns that they describe likely existed prior to current warming. The role of climate change is that it has shifted trees that were already close to their hydraulic limits across survival thresholds, driving an upsurge in tree mortality throughout tree species' ranges and across tree species in a diversity of biomes. As expected from Choat *et al.'s* [48] analyses of widespread hydraulic limitations, climate change-driven tree mortality related to the global increase in frequency and intensity of droughts is currently occurring in range centres as well as at dry range edges and in tropical rain forests as well as in arid regions [33,49–51].

## 3. Drivers of mosaics of climatic stress in Edith's Checkerspot butterfly: results from 50 years of study

Here, using both published and unpublished data and analyses, we illustrate the drivers that have led to different selective forces operating in different ecotypes of the climate-sensitive terrestrial ectotherm *Euphydryas editha* (Edith's Checkerspot butterfly). We describe in detail the eco-evolutionary mechanisms that have caused this insect to deviate from the simple model with which we started, the model in which climatic stress increases gradually towards range margins where it reaches its maxima. Table 1 shows that our study sites are range-central, in that they lie distant from all three range margins: equatorial, poleward and elevational.

**Table 1.** Locations of study sites analysed in this paper and range limits of the species *Euphydryas editha.* The elevational limit is from our own observations. For poleward and equatorial limits, we give two values: uppermost are those we currently find in iNaturalist with correct photographic species verification; below them are the names (Whistler's Peak and El Rosario) and locations of sites at known prior limits used by Parmesan [52] from our own censuses and from private and museum records verified by the authors with a physical specimen. It is not a typographical error that populations at the reported poleward limits are both at much higher elevations than those at the equatorial limits.

category of site	identity of site	latitude	longitude	elevation (m)
poleward limit (1)	iNaturalist limit	52.986	−117.371	1718
poleward limit (2)	Whistler's Peak	52.827	−118.130	2420
equatorial limit (1)	iNaturalist limit	30.027	−115.597	408
equatorial limit (2)	El Rosario	30.161	−115.793	25
elevational limit	Mt Dana summit	37.899	−119.221	3955
Study site, central California	Rabbit Meadow	36.710	−118.373	2380
Study site: central California	Jasper Ridge	37.401	−122.213	408

The spatial pattern of population extinction and persistence through the species' range had caused the mean location of a population to shift polewards and upwards by the early 1990s [52]. However, we see no clear evidence here (table 1) that the range limits themselves have been moving polewards. iNaturalist records from 2005 (equatorial limit) and 2020 (poleward limit) find a slight extension of both limits compared to the earlier study. However, neither Alberta nor Baja California are heavily populated by naturalists and records are sparse in both regions, so we do not conclude that the iNaturalist records in table 1 represent recent colonizations and range extensions.

Relevant traits of the species *E. editha* are described in §3a, while §§3b and 3c focus on the tradeoffs that have caused populations to evolve into strong climatic stress in range-central regions. An un-named subspecies at Rabbit Meadow (table 1) that inhabits sub-alpine elevations at 2000–2500 m on the Western slopes of the Sierra Nevada and feeds principally on the perennial, hemi-parasitic *Pedicularis semibarbata* (Orobanchaceae) is described in §3b. These butterflies encounter a straightforward tradeoff between predator avoidance and thermal stress. Eggs laid on upper leaves of *Pedicularis* would suffer high mortality from incidental predation by grazers. Most individual females reduce this risk to their offspring, laying eggs close to the hot ground where they routinely experience temperatures more than 20°C above those of ambient air and risk exceeding their thermal tolerance limit at around 48°C. The behaviour of positive geotaxis that results in this egg placement is shown in this video link: https://doi.org/10.1371/journal.pbio.1000529.s015.

A second ecotype, the endangered Bay Checkerspot, living in the coastal hills of the San Francisco Bay region and studied at the Jasper Ridge biological reserve operated by Stanford University (table 1) is described in §3c. A tradeoff between maternal fecundity and timing of oviposition has led to the evolution of delayed oviposition and phenological asynchrony between the offspring and their ephemeral annual hosts, *Plantago erecta* (Plantaginaceae) and *Castilleja densiflorus* (Orobanchaceae)*.* In each of four consecutive years (1968–1971), we observed over 90% mortality caused by this asynchrony [53,54], an observation repeated by other authors in subsequent decades [55–57]*.* Since the plants and insects respond differently to climate [58], the evolved asynchrony between them renders this ecotype's population dynamics climate-sensitive, and climatic change was attributed as the principal cause of extinction in 1998 of the long-studied Jasper Ridge metapopulation in its protected habitat [59,60].

As part of §3c, we present a new analysis of our 1970 field dataset from the Bay Checkerspot in which we estimate natural selection operating on phenology at different times during the season. The aim is to understand the observed routine phenological asynchrony between insect and host. In common with most evolutionary biologists (e.g. [61]), we use the concept of natural selection as an effect of phenotype on fitness. Natural selection exists when different phenotypes—in the present case, phenotypes that affect phenology—have different effects on fitness, estimated here from the combinations of female fecundity and offspring survival resulting from particular phenologies. Under this view of selection, it is not necessary to know the source of phenotypic variation—for example, whether it is heritable or plastic. The extent to which a trait is heritable determines the strength of influence that selection has on evolution.

Previously in this journal, using *E. editha* and Winter Moth (*Opheroptera brumata)* as examples, we noted that strong insect–host asynchronies had existed prior to recent climate warming and were likely due to the adaptive evolution of the insects. Therefore, we argued that recent observed asynchronies in other exploiter–victim interactions should not be automatically attributed to anthropogenic climate change [62]. Here, we use the same dataset to investigate natural selection underlying the evolution of phenotypes that experience climatic stress.

In order to render interactions between *E. editha* and its hosts understandable, and to explain the forms of climatic stress that *E. editha* suffers, we describe below the relevant life-history traits of the butterfly and their variability among populations adapted to different hosts and habitats (§3a). We expand and justify our summary (above) of the tradeoffs faced by the two ecotypes, using observations and experiments done across more than five decades (§§3b and 3c).

### (a) Life history and climate-relevant biology of Edith's Checkerspot

Edith's Checkerspot is non-migratory and unusually sedentary for a butterfly [63–66]. This trait has enabled it to evolve extensive local ecotypic variation [67], adapting to meadow, forest, talus and tundra habitats across its range in Western North America that extends from sea level to over 3900 m elevation and from latitude 30° to 53° (table 1). There is a single generation per year, with a short flight period that can be at any season from February to August, depending on habitat. The active stages of the life cycle last 2–5 months, with the remaining 7–10 months spent inactive as part-grown diapausing larvae.

At our Jasper Ridge study site in coastal California south of San Francisco (table 1), diapause of the endangered Bay Checkerspot, *Euphydryas editha bayensis*, typically began in late April or early May and lasted through summer, autumn and early winter until broken by winter rains [62,68]. Post-diapause larvae typically pupated in March, butterflies flew in March–April, eggs hatched in 2–3 weeks and pre-diapause larvae fed for a further 10–20 days.

At our montane Rabbit Meadow study site on the Western slopes of the Sierra Nevada (table 1), diapause is broken at snowmelt, the timing of which is variable among years (from early March to mid-June [69]) and adults fly for 3–6 weeks between late April, and early August, depending on the year. We use a past tense here to describe the Jasper Ridge metapopulation because, as we shall describe, it has been extinct for about 20 years. We use the present tense for Rabbit Meadow because we observed these butterflies to be abundant in 2019 and we are informed that they were still present in 2021 (Matt Murphy and Dennis Murphy 2021, personal communication).

At sites where the hosts of *E. editha* are ephemeral annuals threatening to die next week, the insects are severely time-constrained. At Jasper Ridge, females of *E. e. bayensis* emerged with hundreds of eggs already mature, commenced oviposition on the first or second day of adult life and laid most of their eggs in the first few days of their approximately two-week lifespan [70,71]. By contrast, where the hosts are perennials not threatening to die—for example *Pedicularis* at Rabbit Meadow—the butterflies commence oviposition between days 2 and 4 of adulthood and spread their reproduction more evenly across a similarly brief adult lifespan [72].

#### (i) Important constraint on life history

Butterflies in the subfamily Melitaeinae, to which *E. editha* belongs, cannot diapause as very small larvae; they need to feed through more than two instars before they can rest. This is an odd constraint since several species in different subfamilies of the same butterfly family (Nymphalidae) diapause just after hatching from the egg, surviving through late summer and all of autumn and winter without feeding at all before taking their first bites the next spring [73]. Examples of diapause by neonate larvae exist in the genera *Argynnis, Speyeria, Clossiana* and *Boloria*. *Argynnis paphia* larvae even have to search for their hosts after diapause ends, since the eggs are laid on tree trunks and the hosts, violets, don't grow on tree trunks [73]. By contrast, *E. editha* larvae must feed after eclosion from the egg. If they run out of food before reaching mid-third instar, they search until they find food or starve. After growing to mid-third instar, which takes 10–20 days, they can respond to lack of food by diapause, but if they continue to find food they enter an obligate diapause about halfway through their larval development at the beginning of either the fourth or fifth of six or seven instars [60]. In any case, they do not complete development in the year of their birth.

#### (ii) Effect of egg placement on eggspace temperature

**Figure 2.**
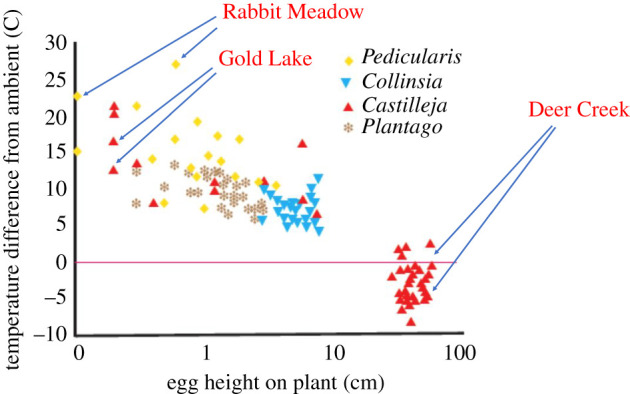
Height above the ground of natural *E. editha* egg clutches related to the difference between eggspace temperatures and temperatures of ambient air at height of one metre. Data were gathered in eight study populations, of which three were monophagous on *Castilleja,* two on *Collinsia* and one on *Plantago*. The remaining two were biphagous, feeding on *Pedicularis* and *Castilleja.* The figure shows individual measures of differences between eggspace temperature and ambient air at 1 m height, with several measures from each population. Symbols indicate the identity of the host genus, not the identity of the population. Details of sites and hosts are in table 3 of Bennett *et al*. [75], from which this figure is modified. The three sites named in the figure are chosen to illustrate descriptions in the text. Arrows show only two of the several readings from each of these three sites and are placed to avoid obscuring data.

Populations of *E. editha* show heritable differences in behavioural geotaxis, affecting the heights above the ground at which eggs are laid and at which larvae feed [67,74]. This height strongly affects the temperature differential between ambient air and the eggspaces adjacent to egg clutches and young larvae (figure 2). The data cover four host genera in eight populations distributed from southern California to southern Oregon and from near sea level to 3171 m elevation [75]. Irrespective of study site or host affiliation, eggs that are closer to the ground experience greater temperature excess over ambient air.

#### (iii) Comparison of egg heights and eggspace temperatures between populations using the same host in hot and cool climates

**Figure 3.**
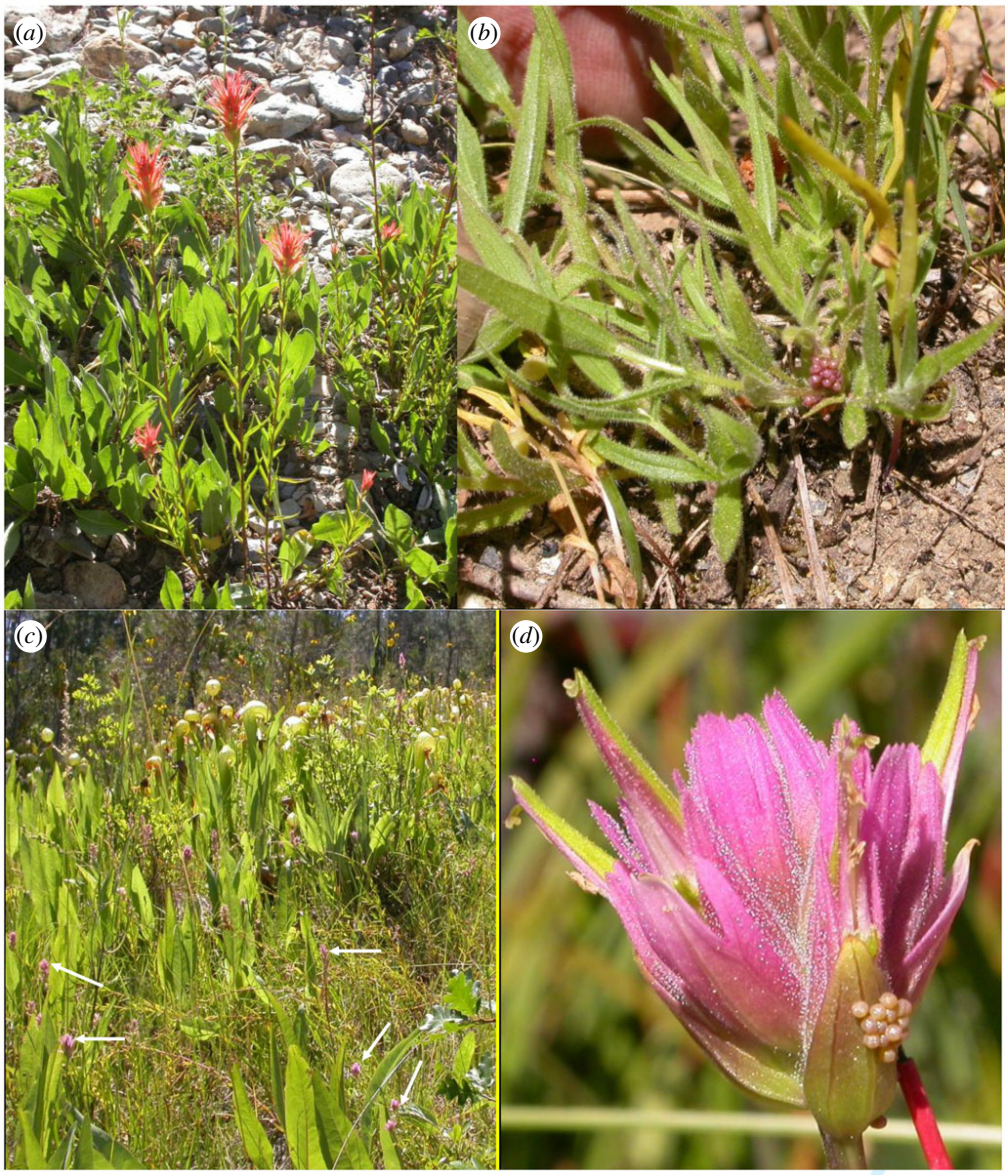
Modulation of eggspace temperatures by choice of oviposition height. (*a*) *Castilleja miniata* at Gold Lake, Amador County, California. (*b*) *E. editha* eggs placed low at Gold Lake and heated by radiation and convection from the ground. The human is tilting the plant away from the camera to show eggs and raising them slightly in doing so. (*c*) *Castilleja miniata elata* at Deer Creek fen, Josephine County, Oregon with *Castilleja* flowers highlighted by white arrows. (*d*) *E. editha* eggs typically placed high at Deer Creek and cooled slightly below ambient air by host transpiration. The rod is the thermocouple used to measure eggspace temperature.

The effect of egg height on eggspace temperature is clearest in a comparison between two sites (table 2) where *E. editha* flies at the same time of year (July) and chooses very different heights on the same host species, *Castilleja miniata* (Orobanchaceae), resulting in a 14°C difference in the relationship between eggspace and ambient temperatures. Ambient temperature at Gold Lake when we made recordings was 10 degrees cooler than at Deer Creek fen, but eggspaces were 4°C hotter. Named arrows on figure 2 lead to separate clusters of points derived from the two study sites. Figure 3 shows the difference in typical egg placement between the two sites and table 2 lists egg heights, ambient and eggspace temperatures.

**Table 2.** Egg heights, ambient temperatures and eggspace temperatures at two sites using the same host species, *Castilleja miniata* (from [75]).

site (number of observations)	elevation (m)	egg height: mean (range) (c m)	mean ambient temperature (°C) during sampling	eggspace temperature: mean (range) (°C)
	latitude			
	longitude			
Deer Creek (34)	elevation 402	45.0 (31–63)	33.3°	30.5° (27.2°–34.8°)
	lat 42.277			
	long −123.648			
Gold Lake (8)	elevation 2073	0.99 (0.4–3.1)	23.4°	34.8° (31.5°–41.7°)
	lat 39.666			
	long −120.675			

### (b) Tradeoffs that cause adaptive evolution into climatic stress: tradeoff number 1, between thermal stress and predation risk

#### (i) Frequent thermal stress caused by oviposition choices

**Figure 4.**
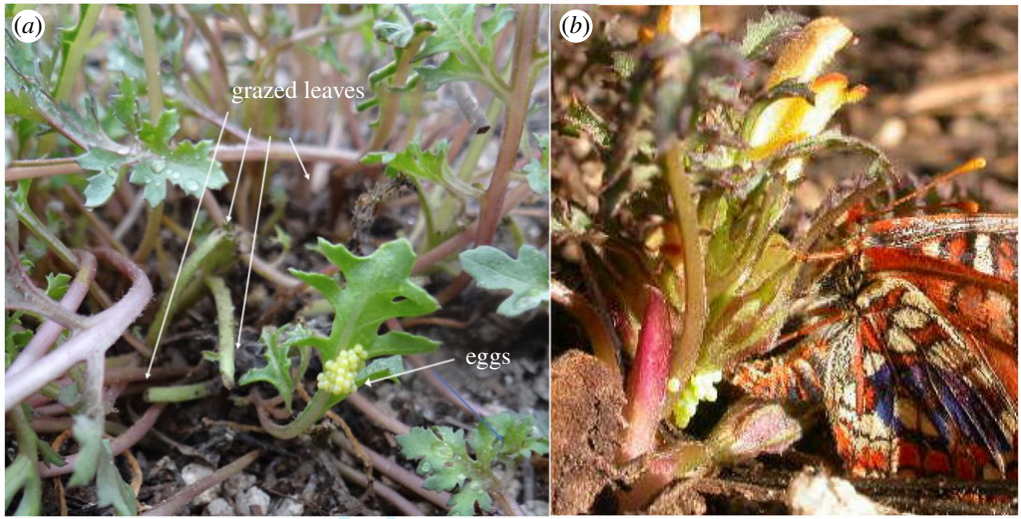
Egg placement in a *Pedicularis-*feeding population at Rabbit Meadow. (*a*) A natural clutch of *E. editha* eggs on *P. semibarbata* at Rabbit Meadow on 25 July 2019, adjacent to grazed leaves. (*b*) A Rabbit Meadow butterfly, numbered ‘6’ with a purple permanent marker, showing positive geotaxis by placing her eggs at the base of a *Pedicularis*.

The population of *E. editha* at Rabbit Meadow in Sequoia National Forest (California) illustrates a straightforward tradeoff in which placement of eggs by ovipositing females exposes them either to incidental predation by grazers or to extreme thermal stress. The butterflies oviposit principally on *Pedicularis*, which is a hemiparasite, requiring root contact with a pine or fir tree in order to survive but also sequestering alkaloids from herbs that it encouters [76]. By some combination of its chemical defenses and association with ants, it is well-defended against grazing vertebrates, which avoid it when alternatives are available in early spring. However, as the California springtime merges into dry summer and the parasitic *Pedicularis* remains juicy while other herbs wilt, grazing on *Pedicularis* intensifies, often simultaneously with peak oviposition by *E. editha* [75,77]. Some whole plants are removed by the grazers, but in most cases, leaves are clipped close to their bases (figure 4*a*). In a dataset pooled from 4 years of observation, just under half the surviving *Pedicularis* plants (*n* = 460) suffered this type of grazing during the season when *E. editha* eggs were being laid and developing [75].

Figure 4*a* shows how grazing causes incidental mortality, illustrating a near-miss at Rabbit Meadow. A clutch of eggs on an intact, ungrazed leaf sits perilously close to the cut petiole ends of four grazed leaves. The colour of the eggs indicates that they were freshly laid, so may have been placed after the leaves were cut. Even so, the photo clearly shows the risks that eggs face when laid at this height above the ground (4 cm).

Figure 2 shows that the lower that eggs are laid, the hotter the microclimates that they experience. At Rabbit Meadow, the microclimate was frequently extreme: working in ambient temperatures of 22°–25°C, we located 22 naturally laid egg clutches on *Pedicularis* and obtained eight eggspace readings of more than 40°C and four of more than 44°C, with one record of 47.1°C [75]. This last reading was close to lethal, since we recorded 100% mortality after exposing eggs from 16 families to 48°C for just 1 hour [75].

The tradeoff is clear: eggs that are laid low risk lethal thermal stress and eggs that are laid high risk equally lethal grazing. Unlike adults and larvae, eggs are trapped in the sites chosen by their mothers, with no means of moving either to cooler or to less risky microhabitats. Their mothers cannot protect them from this simple tradeoff and must choose oviposition sites with a high risk of grazing, high risk of thermal stress, or moderate risk of both.

In practice, at least until recently, most of the eggs at Rabbit Meadow have been laid close to the ground as shown in figure 4*b*; their mean height was reported as 0.55 cm [67]. As we already referenced, the geotactic behaviour pattern that produces this low egg placement, with butterflies dropping to the ground after tasting a leaf and actively searching for the base of the plant is shown in https://doi.org/10.1371/journal.pbio.1000529.s015, a staged encounter between an oviposition-motivated female *E. editha* at Rabbit Meadow and an undisturbed *Pedicularis* growing in its natural position.

The thermal stress that results from this behaviour stems solely from oviposition choices made by females and not from the Rabbit Meadow population living close to a range boundary (table 1) or from a hot macroclimate; the elevation of the site at greater than 2300 m should hold maximum ambient air temperatures below 35°C, given that the recorded local maximum in Fresno at 50 m elevation at the foot of the mountains below Rabbit Meadow is around 47°C.

### (c) Tradeoffs that cause adaptive evolution into climatic stress: tradeoff number 2, between maternal fecundity and offspring mortality

#### (i) The symptom: routine high mortality caused by butterfly–host phenological asynchrony prior to anthropogenic climate warming

*Euphydryas editha* in populations that use ephemeral annual hosts routinely suffer high mortality when host senescence occurs before larvae are large enough to diapause (see §3a(i)). In a small (three-patch) metapopulation of the endangered Bay Checkerspot, *E. editha bayensis,* that inhabited Stanford University's Jasper Ridge reserve until 1998 [59,60], eggs were laid on *P. erecta* and larvae that had fed for a few days had the possibility to move to their secondary host, *C. densiflorus*, if it grew adjacent to the *Plantago* [54,58].

Most larvae starved immediately on hatching from their eggs, without feeding at all, because their hosts senesced during the two weeks between oviposition and egg hatch. The proportions of observed natural clutches suffering complete mortality from this cause were 77% in 1968 and 80% in 1969 [53,54]. Further mortality from the same cause, host senescence, killed most larvae that did start to feed but failed to reach diapause, raising estimated mortality from insect–host phenological asynchrony to around 98% in both 1968 and 1969 [54].

The estimate of 98% pre-diapause mortality from starvation may have been too high, since Hellman [58] showed host-searching abilities of neonate larvae to be greater than Singer [53] had used in estimating survival. Nonetheless, later work at Jasper Ridge recorded routine extreme pre-diapause mortality. White [55] recorded 98–99% mortality in both 1972 and 1973; and in 1983 Dobkin *et al*. [56] documented a strong increase over time of larval mortality from host senescence. In another metapopulation of the same subspecies at Kerby Canyon, Cushman *et al*. [57] estimated an insect–host asynchrony so extreme that there was zero survival from eggs laid in the entire second half of the flight season, while Fleishman *et al*. [78], in a different year, found no penalty for late oviposition. Experimental warming did not increase the insect–host asynchrony and was beneficial to larvae when they were able to use *Castilleja* [58].

**Figure 5.**
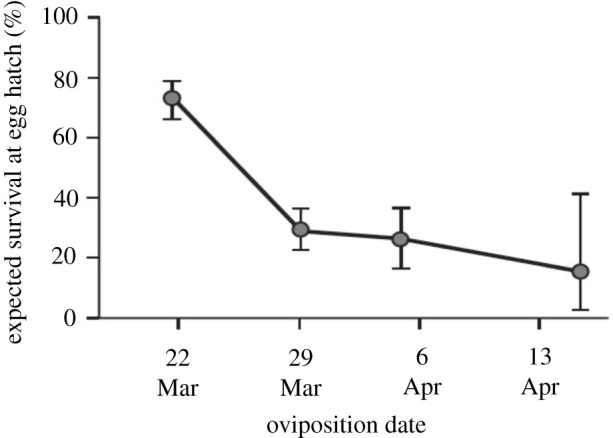
Estimated proportions of *E. editha bayensis* egg clutches at Jasper Ridge in 1970 that would have hatched with access to a non-senescent host after being laid on four dates: 22 and 29 March; 4 and 16 April. Error bars are 95% c.i. Dates indicated on the *x*-axis are spaced one week apart, although the censuses were not performed exactly at this interval; hence, the data shown for 4 and 16 April do not correspond with bars on the *x*-axis. As an example of how calculations were done for each date, the dot above 22 March shows estimated survival at hatching on 4 April of eggs laid on 22 March, using data gathered on both dates on availability and condition of hosts. Estimated survival of eggs laid on 29 March was based on fewer data than the estimate for 22 March since fewer plants were suitable for oviposition on the 29th (modified from [62]).

Following the initial observations of high mortality of naturally laid egg clutches in 1968 and 1969, estimates were made at Jasper Ridge in 1970 of the probabilities that neonate larvae hatching from eggs laid at different times would find food and be able at least to start feeding [53,54]. These estimates, shown in figure 5, were made using repeated surveys of the same 290 randomly chosen points in a habitat patch that contained both level ground and north- and south-facing slopes. At each census, each point was checked for its suitability for oviposition and judged suitable when a circular quadrat of 5 cm radius centred on the point contained more than four green leaves of *P. erecta.* Each point that had been judged suitable for oviposition was later judged in terms of its ability to support the survival of neonate larvae. ‘Survival’ was recorded when a quadrat of 10 cm radius, centred on the same point as the 5 cm quadrat, contained at least one living, edible leaf of host (either *Plantago* or *Castilleja*) around the time when eggs would have hatched. Of the 290 quadrats based on random points, 214 contained sufficient *Plantago* for oviposition, but 33 already contained only senescent hosts on the first day of the study, leaving 181 to be judged suitable and to receive ‘oviposition’ on that first day, March 22 (table 3*a*).

**Table 3.** (*a*): Estimated changes in maternal fecundity and neonate larval mortality of Bay Checkerspots across the 1970 flight season at Jasper Ridge. Time periods are as shown in figure 5. Columns 2 and 3 are the database for calculating the estimates of survival shown in the figure. Column 2: numbers of randomly placed 5 cm radius quadrats judged suitable for oviposition on the dates indicated. Column 3: numbers of 10 cm radius quadrats suitable for neonate larval survival at the second census after ‘oviposition’. Column 4: estimates of % mortality at egg hatch. To clarify, the number 51 in column 3 refers to the number of the 175 micro-sites chosen on 29 March that were suitable for larvae in the 16 April census, around the date when eggs laid on 29 March would hatch. The numbers 25 and 3 in the same column refer to estimated survival at egg hatch on dates later than those shown in the table. The neonate mortality figure of 71% in column 4 is calculated as (175 – 51)/175. At the top of the 5th column is the observed mean fecundity at the beginning of the flight season. Below are expected mean fecundities that would have been achieved by females that could have eclosed to lay 570 eggs on 22 March, but instead chose to continue feeding as larvae on north or south slopes. (*b*) Expected total numbers of neonate offspring surviving per female for individuals delaying development through three specified time periods, calculated from the fecundity and mortality figures in table 3*a*.

(*a*)				
date	number quadrats OK for oviposition	number quadrats OK at egg hatch	estimated neonate mortality	estimated mean fecundity north/south/maximum
22 March	181	132	27%	570
29 March	175	51	71%	634/852/932
4 April	98	25	74%	680/1054/1192
16 April	18	3	83%	790/1538/1814

(*b*)				
delay of oviposition	offspring/female surviving to start feeding NORTH SLOPE MEAN	offspring/female surviving to start feeding SOUTH SLOPE MEAN	offspring/female surviving to start feeding SOUTH SLOPE MAXIMUM	
22 March no delay	416	416	416	
22–29 March	184	247	270	
22 March–4 April	176	274	309	
22 March–16 April	134	261	308	

The result from these calculations (figure 5) is that estimated survival at egg hatch was less than 100% even at the very beginning of the season because many hosts senesced even between the time of earliest oviposition and hatching of the earliest eggs. The probability of survival declined further with later dates of oviposition, so phenological stress from insect–host asynchrony increased over time. However, the figure shows that this stress did not increase as fast as might be expected from the rapidity with which the hosts were dying. The reason lies in the temporal pattern of host senescence: the probability that a plant currently edible and available for oviposition would remain edible until egg hatch declined rapidly for eggs laid between the beginning of the season and one week later (22–29 March), but then changed more slowly through the remainder of the flight period (figure 5).

#### (ii) The tradeoff itself: mothers can choose to be slim and early or fat and late, lateness increases offspring mortality through increased phenological asynchrony of offspring with host

Observations of routinely high larval mortality from host senescence make the insect's phenology seem maladaptive, but from the mother's perspective, it is not necessarily so. If a final-instar female larva that has achieved the minimum size for pupation continues to feed and delays adulthood, she increases her own fecundity as well as her offspring's mortality from host senescence. There is an intergenerational conflict, a tradeoff between maternal fecundity and offspring survival. Typically in such a tradeoff, neither trait is optimized, so we expect neither fecundity nor survival to be maximized.

The fact that offspring survival is not maximized in this case appears as routinely observed high mortality from phenological asynchrony with hosts. The fact that fecundity is not maximized appears as extreme variability; in a sample of 221 newly emerged wild-caught individuals, estimated fecundity ranged from 135 to 1680 [57]. We perceive an analogy with the well-known tradeoff between maternal fecundity and offspring survival stemming from maternal decisions about offspring size: a mother can increase her fecundity by making her offspring small, but usually at the expense of each individual offspring's probability of survival. A female Bay Checkerspot can increase her fecundity by making her offspring late rather than small, but the effect on their survival is the same.

#### (iii) Finding: natural selection on timing in 1970: high fitness of early females followed by plateau as tradeoff allowed different combinations of mortality and fecundity to generate similar fitness values

Here, we expand on prior publications [54,62] by estimating natural selection on butterfly phenology during the three time periods depicted in figure 5 that encompassed the entire flight season in 1970. We show in figure 5 and in column 4 of table 3 how much neonate mortality would be incurred by oviposition on each of the four dates. We then estimate how much fecundity would be gained by delaying maturity and oviposition from the earliest observed date, 22 March, to the next three census dates. We ask to what extent these gains would compensate for the additional mortalities suffered as a result of delaying oviposition to those three dates. To make the fecundity calculations, we use the following data from field measurements and observations:

(1) Mean mass gains per day of last-instar larvae were 8 mg on north-facing slopes, 35 mg on south-facing slopes, reaching a maximum of 45 mg per day under optimum conditions at the end of the larval development season [79].
(2) Mean maximum female larval mass was 400 mg [68]. Mass is lost during pupation; mean mass of newly eclosed female adults was 230 mg [80].
(3) Mean fecundity in a sample of adults emerging at the very start of the flight season was 570 [57].
(4) Each milligram gained by an adult female, above the minimum viable mass, translated into exactly two extra eggs [71].
(5) Experimental warming accelerated insect development slightly more than host development, thereby improving larval survival despite advancing host senescence [58].

Using these data, we set the fecundity at the start of the flight season to 570 and calculated how much additional mass would be gained by postponing pupation and continuing to feed on warm and cool slopes. We then translated that additional mass into additional fecundity over and above the starting value of 570.

Tables 3*a* and 3*b* show that butterflies eclosing at the very start of the flight season would have lost fitness by delaying maturity. Losses on north-facing slopes would have been heaviest. However, at least under the best conditions (south slope maximum), the three different lengths of delay generated similar expectations of fitness because changes in fecundity and mortality compensated for each other.

## 4. Discussion

Sixty years of detailed ecological and evolutionary studies of wild populations of Edith's Checkerspot butterflies across a wide range of habitats and ecotypes have provided a rich source of natural and planned experiments that elucidate both proximate mechanisms and underlying selective drivers of the spatial patterns of climatic stress that we observe. Populations far from the range limits have independently evolved to the limits of their climatic tolerances and rendered themselves climate change-vulnerable by adaptive evolutionary responses to a geographic mosaic of natural selection acting on tradeoffs between climate adaptation and other fitness traits, namely fecundity and predator avoidance.

*E. editha* is sedentary [63,66] and prone to local adaptation over short distances [67], causing climate change-vulnerable populations to exist interspersed with others that are not climate-stressed. Because of this diversity, we have predicted that, at the species level, the insect should be resilient to climate change [75]. Indeed, the demographic responses of *E. editha* to the California drought of the late 1970s were diverse: several populations were driven to extinction while others prospered [81].

Species with less local adaptation than *E. editha* may prove more vulnerable to environmental change. For example, if the tradeoff between predator avoidance and thermal stress that we described at Rabbit Meadow (§3b) were to occur homogeneously across a large part of a species' range, then that portion of the range could be rendered homogeneously vulnerable, in contrast with the mosaic of vulnerability that we do see in *E. editha*.

### (a) Evolution to the limits of climatic tolerance increases vulnerability to climate change

We might expect that populations inhabiting naturally fluctuating environments should adapt to tolerate deviations in both directions from the average conditions that they experience, thereby minimizing vulnerability to change and reducing risk of extinction. That is not what the trees studied by Choat *et al*. [48] have done and it is not what Edith's Checkerspot butterfly has done in the two metapopulations we describe here. Instead, both the trees and the butterflies have evolved in different routes to the limits of their climatic tolerances, rendering themselves resilient to change in one direction but vulnerable to change in the other.

Edith's Checkerspot in the Jasper Ridge metapopulation had, prior to its extinction, evolved a life history in which the majority of young larvae routinely died from starvation from failing to fit their life cycles into the available time (table 3; [53–55,57,68,79,82]). Climate change that exacerbates this phenological asynchrony between plants and insects should be strongly detrimental to the butterflies, while change that reduces the asynchrony would be expected to cause population booms.

Inter-year variability of phenological synchrony between Bay Checkerpsots and their hosts is expected, since plants and insects respond differently to weather and climate. In late winter, the black, themophilic *Euphydryas* larvae at Jasper Ridge could heat up even on cold days if the sun shone, speeding their development and gaining time relative to the life cycles of their hosts [68,79,82]. Conversely, the hosts continued to develop slowly in dull wet weather and could gain on the insects on days when the caterpillars could not feed at all. Not surprisingly, from the beginning of the series of studies on Bay Checkerspot, insect–host phonological synchrony was recorded as varying among years [53,54,59,60,68,79,82,83]. Modelling of Checkerspot responses to climate attributed the extinction of the Jasper Ridge metapopulation in 1998 to a series of cloudy springs as well as to increasing inter-year variability of mortality from the effects on insect–host phenological asynchrony of climatic fluctuations associated with warming [59,60,83].

With the additional knowledge we lay out here, we can describe the Jasper Ridge extinction as resulting from the interaction between current climate change and prior adaptive evolution of the insect to high fecundity, with consequent extreme phenological asynchrony with its host. The Bay Checkerspot has also not been helped by the general degradation of San Francisco Bay area habitats with urban sprawl. The hosts at Jasper Ridge, *Plantago erecta* and *Castilleja densiflorus* have suffered increasing competition from invasives encouraged by nitrogen loading from car emissions, and the habitat has, despite its protected status, become degraded since early studies in the 1960s [84].

### (b) Differences between sites in population regulation and vulnerability to climate change

The Jasper Ridge metapopulation is long gone, along with the majority of other populations of Bay Checkerspot, but the montane Rabbit Meadow metapopulation persists in 2021 despite its apparent vulnerability to thermal stress (Matt Murphy and Dennis Murphy 2021, personal communication). There is good reason to expect Rabbit Meadow to be buffered against impacts of changing climate, since intraspecific competition for food has been consistently present over 30 years of study [85,86]. Averaged over many years, mean clutch size of eggs has been 41 [67]. A single *Pedicularis* plant cannot support more than two or three such clutches, yet each year that we have worked at this site between 1978 and 2019 eggs have been nonrandomly distributed and a few individual plants have accumulated many times the number of caterpillars that they could support to diapause [85,86]. In 2019, we found one *Pedicularis* plant with nine egg clutches and another with seven. The majority of larvae hatching on these intensely attacked plants died in intense scramble competition before reaching diapause ([85,86], see also [55]). This history of strong density-dependent mortality predicts that, if egg mortality were to rise due to increasing thermal stress from climate warming, ‘demographic compensation’ [87] would operate, increasing survival of those groups of larvae that survived the egg stage and protecting the metapopulation from climate change-driven extinction.

By contrast, Jasper Ridge had no such regulatory buffer against fluctuations caused by climate change [53,54] and no means of demographic compensation for increasing climate-caused mortality. We have already described (table 3*a*) how most larvae starved without feeding at all, as they hatched from their eggs into an environment where hosts had already senesced. Further mortality of larvae that did start to feed (§3c) was also almost entirely due to host senescence, not to feeding by other larvae. There was almost no density dependence operating at this stage in the Bay Checkespot, and little room for important density dependence later in the life cycle, since mortality at the pre-diapause stage reduced larval density per square metre to approximately the density of adults that would emerge in the following year [53]. Population density at Jasper Ridge was regulated weakly, if at all. Mclaughlin *et al.* [60, p. 538] described the decline of this metapopulation thus: ‘the routes to extinction for *E. e. bayensis* in protected habitat were random walks driven by climatic variability’.

### (c) Tradeoffs facilitate maintenance of variability in climate-sensitive fitness traits

**Figure 6.**
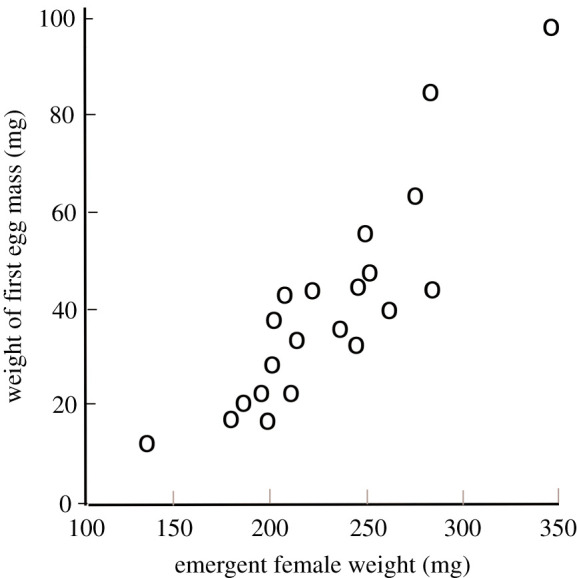
Mass of first egg clutches, plotted against mass of teneral (newly emerged) Bay Checkerspot females (adapted from [80].

Observations throughout the natural world of high variability in heritable traits with strong effects on fitness puzzled evolutionary biologists until they recognized both the ubiquity of tradeoffs and the ability of those tradeoffs to render different strategies equivalent in their effects on fitness [88]. Fecundity of female *E. editha* at Jasper Ridge, itself a key fitness trait and a key component of the tradeoff influencing evolution of phenology, was highly variable. Labine [70] measured fecundities in a sample of 58 field-gathered females and obtained a mean value of 731 with a maximum of 1200, a higher number than had been recorded at that time in any butterfly. Later, Cushman *et al*. [57] recorded an even higher upper limit, estimating a mean of 500 with a range from 135 to 1680. Murphy *et al*. [80] found equivalent variability in laboratory-measured weights and fecundities of newly emerged females that had developed in the field as larvae (figure 6).

Why such variability in an important fitness trait? For *E. e bayensis,* in the year we analysed (table 3*b*), variation along the axis of the fecundity/mortality tradeoff was close to neutral across three-quarters of the flight season, including the time of peak emergence at the beginning of April. This, with the likely addition of plasticity and bet-hedging, would help explain the high variability of female size and fecundity ([70,80], figure 6).

**Figure 7.**
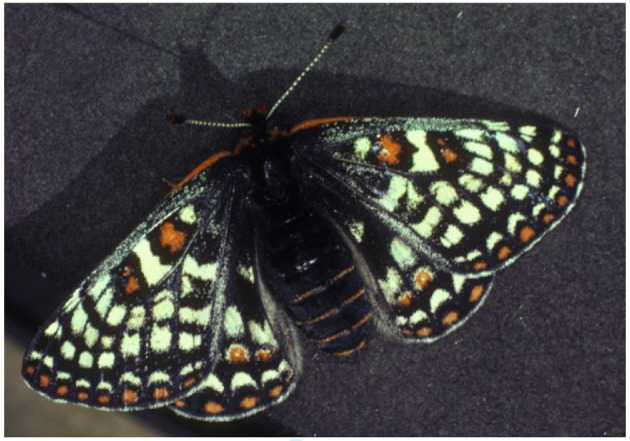
‘Bumblebee-plump’ Bay Checkerspot female distended with eggs prior to laying first clutch.

As Labine [70] first noted and Boggs [71,89] confirmed, the individual Bay Checkerspots with the highest fecundities were extreme among butterflies. We illustrate one of them in figure 7. This newly eclosed, bumblebee-plump female has gained mass and fecundity by delaying emergence. Labine described such individuals as ‘heavy with eggs and restricted in mobility’. This was a polite way to describe these butterflies that, in our own observations, needed to flap hard in order to take off, flew in straight lines once airborne, hardly able to change direction or escape pursuing males, and finally crashed clumsily into the vegetation on landing. However, after laying 2 or 3 egg clutches in the first few days of adult life they quickly became more manoeuvrable, while males underwent the reverse transformation.

In the densest populations of *E. e. bayensis,* while females were becoming more agile as they aged, we observed that males were becoming less able to fly, wearing their wings shorter by frequent fighting. The results were comedic: late in the flight season the females, no longer ‘heavy with eggs’, glided across their habitat while dozens of males trying to follow them jumped vertically into the air flapping their wing-stubs furiously but making no lateral progress and ending up almost exactly where they had started (MC Singer 1969, personal observations).

Overall, the nature of the tradeoff between maternal fecundity and offspring mortality allows for the maintenance of genetic variation in the population which, we argue, provides options for coping with anthropogentic climate change through evolution.

### (d) Plasticity generates phenotypic covariance between fecundity and timing opposite in direction to genetic covariance

Genetic variation in size and fecundity of Edith's Checkerspot has not been conclusively shown, but can be inferred from maintenance in a laboratory culture of the strong interpopulation differences, from mean initial female mass of less than 100 mg to more than 250 mg ([75], see below). To the extent that such variations were to exist within populations, it would generate the expectation that more fecund females emerge later. Females with high-fecundity genotypes would need to spend more time in development.

What is observed in nature is the opposite of the expected covariance between size and timing. In both the Bay Checkerspot [57] and at our Rabbit Meadow study site [90], late-emerging females were significantly smaller than early ones. A clue to the cause lies in the difference between males and females in timing at Rabbit Meadow. In 18 of 20 years, the last insect observed to eclose was male; in one year, the last insect was female and the remaining year was a tie. Conversely, in 8 of 9 years, the earliest insect was male and in only one was it female. These observations suggested that female larvae suffering environmental resistance that rendered them late were surrendering some of their final size to regain part of the lost time, while males were not making the same trade. The hypothesis was supported: late males were not smaller than early ones and the time-trends of size differed significantly between the sexes [90].

Our conclusion is that the observed covariance between fecundity and timing in Edith's Checkerspot, with late females being smaller and less fecund, is not paradoxical, it reflects the dominance of environmental over genetic influences. At Rabbit Meadow, strong environmental variance results from patchy snowmelt in spring, since larvae can only start to feed when released from their snow cover. In the Bay Checkerspot, strong variance results both from topographic diversity [54,68,79,82] and from variation of size at diapause. If food disappears for a larva that has reached mid-third instar, it will moult into its water-resistant diapause phenotype and become a small fourth-instar individual. If it reaches the end of third instar and still has food, it can moult into diapause as a larger-size fourth-instar or bet-hedge and postpone diapause by one instar, feeding in fourth instar and entering diapause in fifth. This postponement is a risky strategy because a larva that has decided to feed in fourth instar must do so at least for a few meals and will starve if its hosts disappear during the 2–3 days while it is waiting to moult and then hardening its jaws.

The result of this complexity, plus the ability of larvae to postpone adulthood, diapause more than once and live several years, is that Bay Checkerspot larvae emerge from diapause in December/January at differing sizes and with differing potentials for achieving high fecundity or early emergence as adults. Weights of 70 diapausing larvae gathered at the same time in 1969 mostly lay between 3.0 and 4.5 mg, typical of fourth-instar diapause. However, four of them weighed between 9.0 and 16.5 mg and had a much greater range of options for combinations of size and timing as adults [53].

### (e) Options for *in situ* adaptive response of Checkerspot populations to changing climate

A sedentary lifestyle combined with strong local adaptation provides a tapestry of genetic variation, more generally than the explicit examples above, which may allow persistence of populations in the face of rapid climate change. Gene flow among Edith's Checkerspot populations is sufficiently low [91] that complex suites of host-adaptive traits can evolve locally and that populations less than 60 km apart can have non-overlapping phenotypes [67,69]. Substantial heritable differentiation of host preference was recorded over a 12 km distance in response to spatially variable natural selection, and over 150 m in response to a combination of natural selection and biased gene flow [77,92].

This ability to adapt locally implies that Checkerspot populations have options for adapting *in situ* to changing climate although, as we have shown, natural selection will not necessarily favour those options. Thermal stress on eggs could be reduced by raising egg heights. Phenological stress from host senescence could be reduced by host-shifting to a longer-lived host or by shortening developmental time (either by increasing egg size or decreasing adult size). Egg size and oviposition preference show heritable variation in *E. editha* and are capable of responding to changes in natural and human-driven selection [72,93]. Indeed, one large population under strong selection to change hosts took only 23 generations to evolve from near-monophagy on its traditional host to complete monophagy on an exotic novel host [94].

Frequently, in nature, evolutionary responses to environmental change are impeded by species-specific constraints. We noted in §3a that the failure of Checkerspot larvae to evolve the ability to diapause without feeding constitutes an important constraint, the lifting of which could completely eliminate the mortality that these larvae currently suffer from host senescence. By contrast, as the following sections show, two other likely constraints, those on size and on host specificity, are not likely to impede the butterflies' adaptation to warming climate.

#### (i) Rejection of adult size as constraint to climate adaptation

We have argued that female Bay Checkspots evolved their heavyweight physique as a result of the fecundity/mortality tradeoff and the pattern in time of host senescence (table 2). However, an alternative reason could be that the size of the insect is constrained and that smaller size and earlier phenology are hard to achieve. We reject this alternative, first because of the high variability of size within the Jasper Ridge population of Bay Checkerspot (figure 6) and, second, because the mean masses of teneral (newly eclosed) females in four *E. editha* populations were recorded by Bennett *et al*. [75] as 92 mg, 131 mg, 182 mg and 285 mg, with the largest number, 285 mg, being from the Kerby Canyon (Morgan Hill) metapopulation of Bay Checkerspot. The Jasper Ridge females weighed by Murphy *et al*. [80] had a mean weight of 224 mg (figure 6). Therefore, Bay Checkerspots had the opportunity to respond to insect–host phenological asynchrony and high mortality of late larvae by evolving to smaller adult size with shorter developmental time, a phenotype that already exists within the species. However, prior to their extinction, the Jasper Ridge butterflies did not avail themselves of that opportunity.

#### (ii) Rejection of host specificity as constraint to climate adaptation: host-shifting is easy

We have observed host shifts in both directions between long-lived perennial and short-lived annual hosts. In one case, anthropogenic fertilization by fire extended the lifespan of the ephemeral *Collinsia* at Rabbit Meadow and triggered an evolutionary host shift from *Pedicularis* to *Collinsia* in the 1970s and 1980s that was reversed in the 1990s as the effects of fertilization diminished and natural selection reverted to favouring oviposition on *Pedicularis* [77]. As described in our account here, the current host of these butterflies is, once again, *Pedicularis*.

In a second case, a long-lived exotic Plantago, *P. lanceolata,* arrived in a population of *E. editha* at Schenider's Meadow (Carson City, Nevada). The butterflies were feeding on *Collinsia* and suffering the same type of asynchrony that we describe for Jasper Ridge. Individuals using the novel host instantly acquired higher fitness, since they were released from the phenological stress associated with using *Collinsia.* Between 1982 and 2005, the population rapidly evolved from only 5% preferring *Plantago* to invariant oviposition preference and monophagy on this novel host. Later, when humans applied a change of land management, removal of cattle, the *Plantago* became overgrown and the butterflies went extinct. Four years later, a natural recolonization returned the diet to *Collinsia* [94].

The rapid evolutionary host shifts we describe here have all occurred following changes of natural selection on diet, brought about by environmental change. Therefore, if climate change alters natural selection on host preference of Edith's Checkerspot, we expect the butterflies to respond rapidly and adaptively. However, host shifts do not necessarily require the evolution of preference; in apparent response to climate warming, the endangered Quino Checkerspot, *Euphydryas editha quino*, has colonized higher elevations than its prior upper elevational limit. This colonization required a host shift but no change of preference was detected in the novel habitat [95].

### (f) Relevance of climate-stress mosaics, local adaptation and evolutionary potential for conservation planning and policy

Range contractions at warm margins indicate that there are limits to species' ranges [87,96] and that often these limits represent boundaries of climatic space beyond which species cannot maintain populations for long periods. Here, we show that these climatic boundaries are not confined to range margins. In our introduction, we reviewed evidence for this pattern in intertidal systems, trees in diverse habitats and yellow warblers. Whether or not the overall climate is changing, these studies across diverse taxa show that range centres can be mosaics of climatic stress with many interior populations operating just as close to fundamental species-specific physiological limits as those at receding range margins, and so just as vulnerable to global climate change. What are the implications of this under-studied and underestimated pattern of climate stress for conservation policy and planning?

Our long-term study of Edith's Checkerspot gives a detailed example of the complexity of the influence of climate, and hence of climate change, on a wild species with a penchant for ecotypic variation of climate-relevant traits. Even though most of those traits are not tightly constrained as such, they are, as we have shown, linked together in ways that create tradeoffs among them and constrain the evolution of optimal trait-combinations. For example, phenology and size cannot both be optimized: large and fecund butterflies are constrained to delayed phenology and asynchrony with ephemeral hosts. Increasing egg height protects from thermal stress but slows development and incurs incidental mortality from grazers, where grazing is common.

Despite these complexities and constraints, the diversity of variable traits that affect Edith's Checkerspot's experience of climate opens up a suite of avenues for *in situ* adaptation, raising the likelihood that populations will persist in the face of climate change, at least into the near-term. This is particularly pertinent as there are now three named subspecies of Edith's Checkerspot on the United States endangered species list: the Bay Checkerspot *(E. e. bayensis), the Quino Checkerspot (E. e. quino)* and Taylor's Checkerspot *(E. e. taylori),* each occupying a distinct habitat type and with its own unique behaviour and ecology.

The existence of range-wide climate stress mosaics such as the one we describe here and those already documented in other taxa [44,48] leads to important policy implications:

(1) Populations that appear to be at high risk from climate change may nonetheless resist extinction, making it worthwhile to continue to protect them, reduce other stressors and monitor for adaptive responses.
(2) Protection and efforts at restoration should not be restricted to equatorial/lower range boundaries—many interior populations may be just as vulnerable as those near the range boundary.
(3) Observation of populations adapting to mitigate climatic stress [97] may suggest mimicking those adaptations as a conservation strategy. A successful example of just such a solution comes from Edith's Checkerspot. Knowing that one population of *E. editha* had escaped from phenological stress and raised fitness by host-shifting from the ephemeral *Collinsia parviflora* to the exotic, long-lived *Plantago lanceolata* [94,98], conservationists have recently re-introduced the endangered Bay Checkerspot to a long-extinct site in San Francisco where anthropogenic influences had reduced availability of the traditional host, *Plantago erecta*, but where the exotic perennial *P. lanceolata* had arrived in abundance [99].
(4) Knowledge of ecotypic variation that has evolved in the context of particular climate regimes may suggest strategies for restoration and for aiding vulnerable populations to adapt *in situ*, including selective introductions of key genotypes as ‘genetic rescue’.
(5) In the face of uncertainties in future climate change, coupled with limited understanding of complex selective forces and responses in wild populations, the best option for conservation planning and management is to preserve phenotypic and genetic diversity at every level of biological organization—from populations, to species, to communities to phylogenetic diversity. This superficially sounds like a call to ‘save everything’, but it is not. It means that, when considering options for development and protected area planning, it is imperative to have multi-level biological diversity as a key target for that decision process.

In sum, our results point to the need for a better understanding of the interplay between events at range limits and range centres so that knowledge of the eco-evolutionary dynamics shaping climate stresses across the ranges of wild species will complement the increasingly sophisticated process-based eco–evo models [100] and help forecast the ways in which interacting species and communities will change in response to environmental changes in the coming decades [101].

Notably, even in wild, undisturbed populations, natural constraints to the expression of climate-adaptive traits may exist that impact the ultimate selection forces at play, and hence the ability of that population to cope with changing climate. This is even more true for systems with additional human stressors (e.g. invasives, nitrogen addition, pollution and habitat fragmenation). Non-climatic anthropogenic drivers modify historical constraints, making future projections of species' risk from anthropogenic climate change challenging. Conservation in a time of rapid climate change will require flexible, adaptive planning and management and perhaps adjusting goals to more strongly emphasize the preservation of diversity in a broad context across species, communities, and regions.

## Data accessibility

Data are presented in the paper itself and in the electronic supplementary material [102].
